# Intracardiac Migration of an Iliac Venous Stent to the Right Ventricle Causing Tricuspid Regurgitation Managed by Surgical Extraction: A Case Report

**DOI:** 10.7759/cureus.113651

**Published:** 2026-07-30

**Authors:** Driss Britel, Emane Mengue Phalex, Soumia Faid, Mehdi Moujahid, Zouhair Lakhal

**Affiliations:** 1 Cardiac Catheterization Unit, Mohammed V Military Hospital, Rabat, MAR; 2 Cardiac Intensive Care Unit, Mohammed V Military Hospital, Rabat, MAR

**Keywords:** iliac venous stent, open-heart surgery, right ventricule, stent migration, tricuspid regurgitation, venous stenting

## Abstract

Venous stenting is increasingly used in the management of chronic obstructive venous disease, particularly in severe post-thrombotic syndrome, iliocaval obstruction, and extrinsic iliac vein compression. Although this technique is generally effective and safe, rare but potentially serious complications may occur, including stent migration to the right-sided cardiac chambers.

We report the case of a 72-year-old man with a history of deep venous thrombosis treated by left iliac venous stenting approximately 20 years earlier. He presented with a cough and hemoptysis evolving over three months. Chest computed tomography angiography revealed a suspicious right basal pulmonary lesion, which remained under investigation, and an intracardiac foreign body corresponding to a migrated venous stent within the right-sided cardiac chambers. On admission to the intensive care unit, the patient had clinical signs of right-sided heart failure. Transthoracic echocardiography confirmed a stent straddling the right atrium and right ventricle, in contact with the septal leaflet of the tricuspid valve and causing moderate tricuspid regurgitation. Because of its entanglement with the tricuspid valve apparatus, endovascular retrieval was considered unsuitable. The patient underwent surgical extraction through median sternotomy under cardiopulmonary bypass. The postoperative course was uneventful, and the patient was discharged on postoperative day nine. At three-week follow-up, he remained asymptomatic and clinically stable.

Intracardiac migration of a venous stent is rare but may lead to severe complications, including valvular injury, arrhythmia, embolization, perforation, and heart failure. This case highlights the importance of appropriate stent sizing, careful deployment, and long-term clinical and imaging surveillance after venous stenting.

## Introduction

Venous stenting has become an important therapeutic option for selected patients with chronic obstructive venous disease, particularly in cases of severe post-thrombotic syndrome, iliocaval obstruction, and extrinsic iliac vein compression such as May-Thurner syndrome [1,2]. According to the 2020 appropriate use criteria, iliac vein or inferior vena cava stenting may be considered appropriate in selected symptomatic patients with advanced chronic venous disease, particularly those classified as Clinical, Etiological, Anatomical, and Pathophysiological (CEAP) C4-C6, in the absence of significant superficial venous reflux and when intravascular ultrasound demonstrates at least 50% iliac or caval stenosis or occlusion [3]. By restoring venous patency, this procedure can reduce venous hypertension, improve symptoms, and enhance quality of life in appropriately selected patients [2,3].

Although venous stenting is generally effective, it is not free from complications. Reported adverse events include in-stent restenosis, thrombosis, infection, fracture, and, more rarely, stent migration [4,5]. Migration of a venous stent to the right-sided cardiac chambers is an uncommon but potentially serious complication [5-8]. Depending on the final location of the migrated device, patients may remain asymptomatic or may develop complications such as arrhythmia, tricuspid valve dysfunction, right-sided heart failure, cardiac perforation, endocarditis, or pulmonary embolization [6-8].

We report the case of a 72-year-old man with a history of left iliac venous stenting for deep venous thrombosis who was incidentally found to have intracardiac migration of the stent into the right atrium and right ventricle. The migrated stent was in contact with the tricuspid valve apparatus and caused moderate tricuspid regurgitation, requiring open-heart surgical extraction.

## Case presentation

A 72-year-old man was referred to our institution for evaluation of chronic cough and hemoptysis that had evolved over three months. His medical history was notable for deep venous thrombosis treated approximately 20 years earlier with left iliac vein stent placement. He also had cardiovascular risk factors, including advanced age, male sex, and a history of chronic smoking estimated at 50 pack-years, which he had discontinued 15 years before presentation.

**Figure 1:**
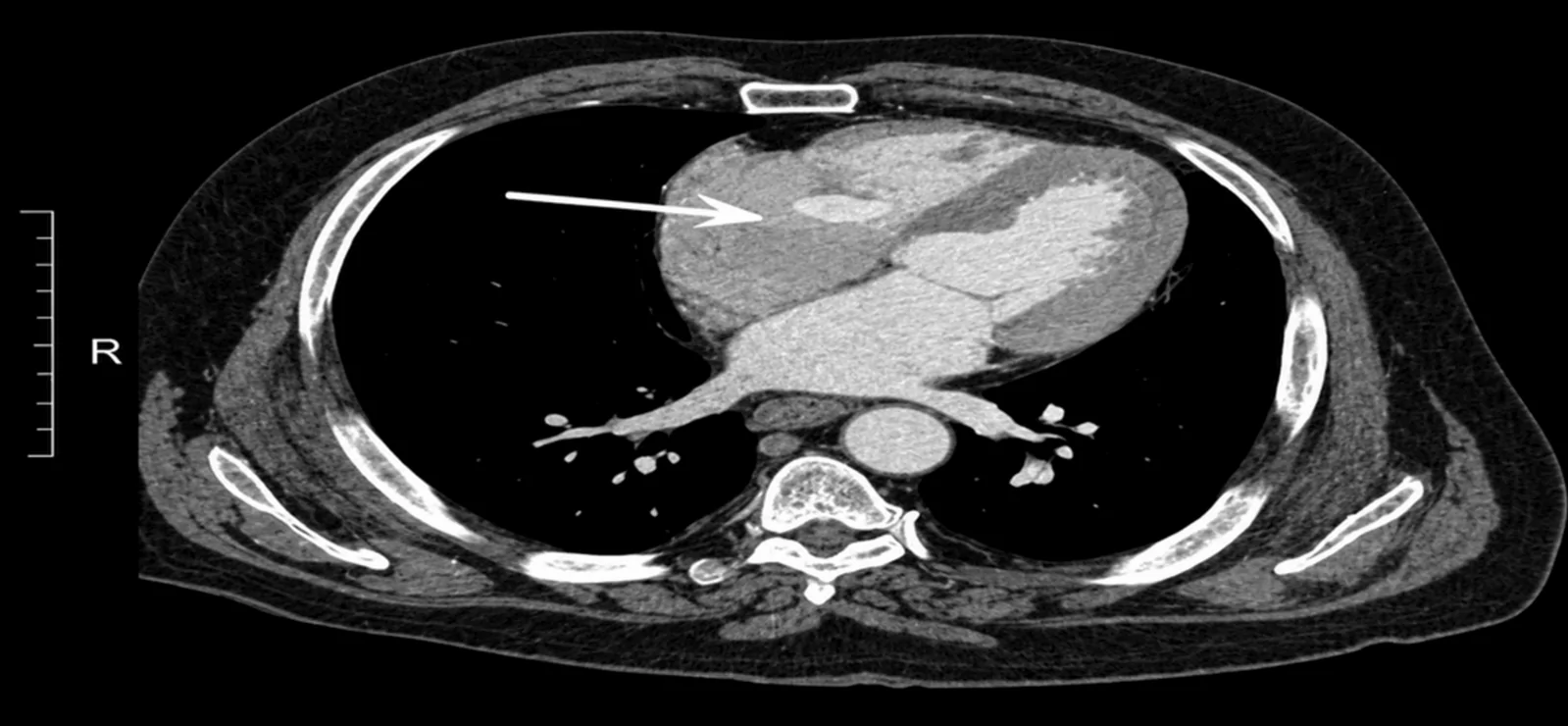
Chest computed tomography angiography showing a migrated venous stent within the right-sided cardiac chambers. The arrow indicates the migrated stent projecting across the right atrium and right ventricle.

The patient initially consulted in the private pulmonology sector, where imaging revealed a suspicious pulmonary lesion that had not yet been fully characterized. He was subsequently referred to the pulmonology department of Mohammed V Military Teaching Hospital, Rabat, Morocco. Because of persistent respiratory symptoms and newly developed exertional dyspnea, chest computed tomography angiography was performed. The scan showed a suspicious right basal pulmonary lesion, which remained under investigation, and incidentally revealed a migrated stent within the right-sided cardiac chambers (Figure 1). The patient was then transferred to the cardiac intensive care unit for preoperative assessment and multidisciplinary management.

On admission, the patient was slightly tachypneic but tolerated the supine position. His oxygen saturation was 96% on room air, and his respiratory rate was 29 breaths per minute. He reported mild lower-limb edema. Physical examination revealed jugular venous distension and a positive hepatojugular reflux. Pulmonary auscultation did not reveal rales, and cardiac auscultation did not detect any additional heart sounds or obvious murmur. The patient was hemodynamically stable, with a blood pressure of 120/80 mmHg and a heart rate of 75 beats per minute. Neurological examination was normal, with a Glasgow Coma Scale score of 15/15.

The principal laboratory findings at admission and discharge are summarized in Table 1. All reported values were within the corresponding reference ranges.

**Table 1:** Principal laboratory parameters measured at hospital admission and at discharge, with the corresponding institutional reference ranges. All reported values were within the reference ranges.

Laboratory parameter	Admission value	Discharge value	Reference range
Hemoglobin, g/dL	14	13	13.0-17.0
White blood cell count, ×10⁹/L	7.8	6.5	4.0-10.0
Platelet count, ×10⁹/L	160	210	150-400
Serum creatinine, mg/dL	0.9	1.1	0.7-1.3
Blood urea nitrogen, mg/dL	8	7	7-20
Sodium, mmol/L	136	138	135-145
Potassium, mmol/L	3.9	4.2	3.5-5.0
C-reactive protein, mg/L	1.2	2.7	<5
Prothrombin time/ international normalised ratio	1	0.9	0.8-1.2
Activated partial thromboplastin time (seconds)	27	30	25-35

**Figure 2:**
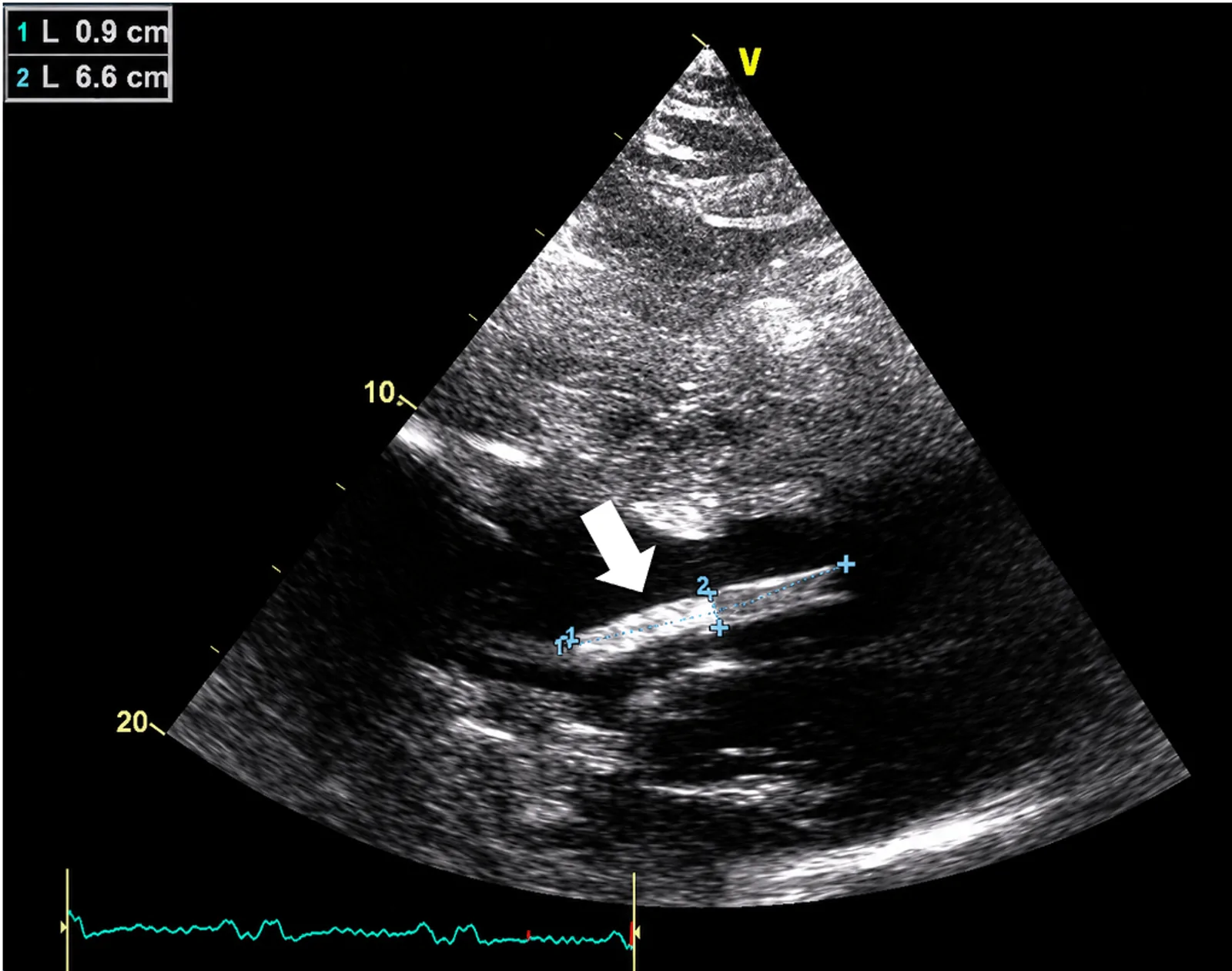
Transthoracic echocardiography showing a linear echogenic foreign body within the right-sided cardiac chambers, consistent with a migrated venous stent measuring approximately 66 × 9 mm. The arrow indicates the migrated stent.

**Figure 3:**
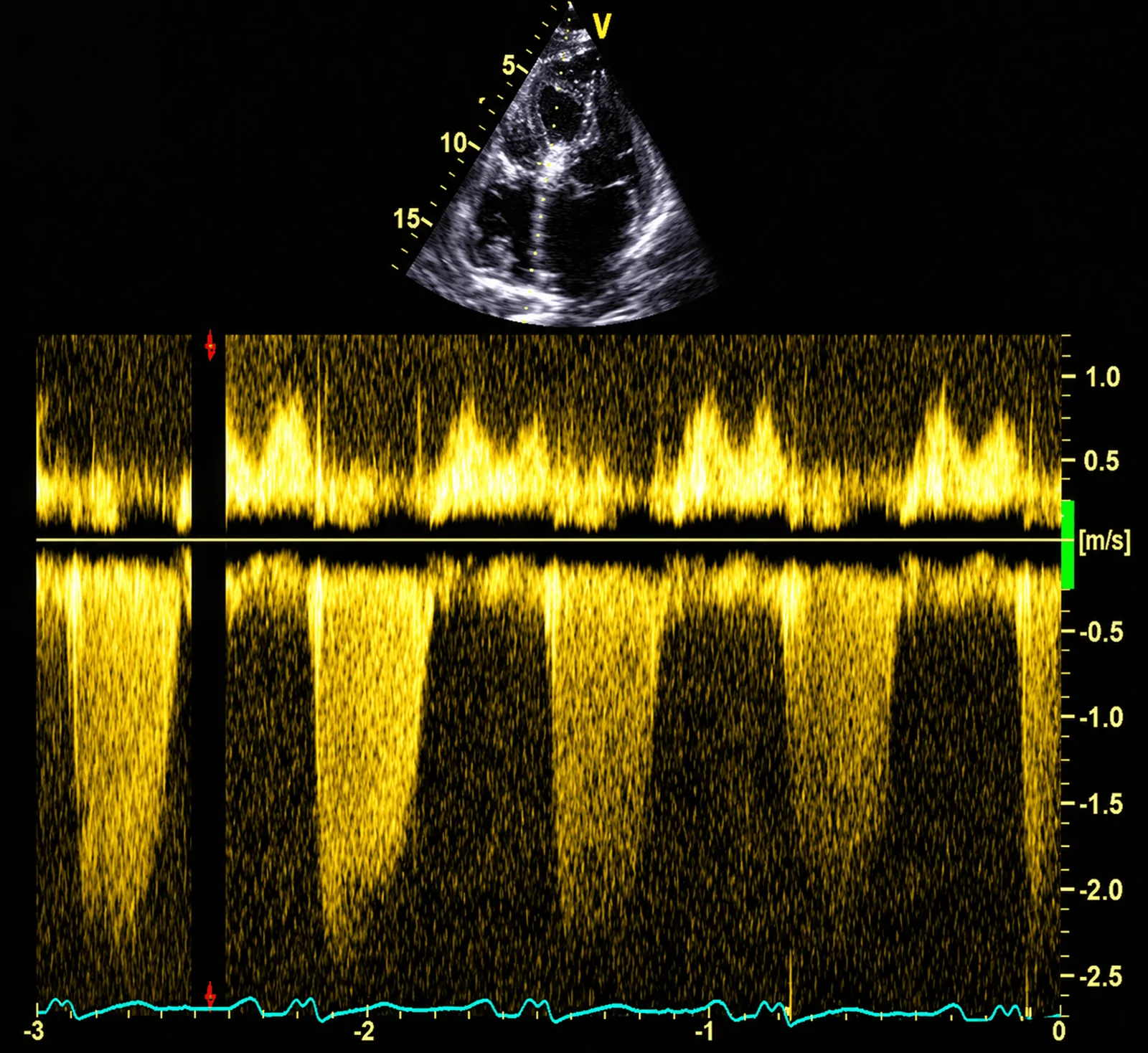
Doppler assessment of tricuspid regurgitation Continuous-wave Doppler transthoracic echocardiography showing moderate tricuspid regurgitation secondary to interaction between the migrated venous stent and the tricuspid valve apparatus. The maximal tricuspid regurgitation velocity was estimated at 2.7 m/s, with an estimated systolic pulmonary artery pressure of approximately 30 mmHg.

The electrocardiogram showed no significant abnormalities. Transthoracic echocardiography confirmed the presence of a linear intracardiac foreign body consistent with a migrated venous stent, extending from the right atrium to the right ventricle. The stent measured approximately 66 × 9 mm and was in contact with the septal leaflet of the tricuspid valve (Figure 2). There was no evidence of right ventricular outflow tract obstruction. The right atrium was dilated, with an estimated area of 25 cm². Doppler evaluation showed moderate tricuspid regurgitation, with a maximal tricuspid regurgitation velocity of 2.7 m/s, and an estimated systolic pulmonary artery pressure of approximately 30 mmHg (Figure 3). Coronary angiography was performed as part of the preoperative assessment and showed coronary arteries of good caliber without significant stenosis.

**Figure 4:**
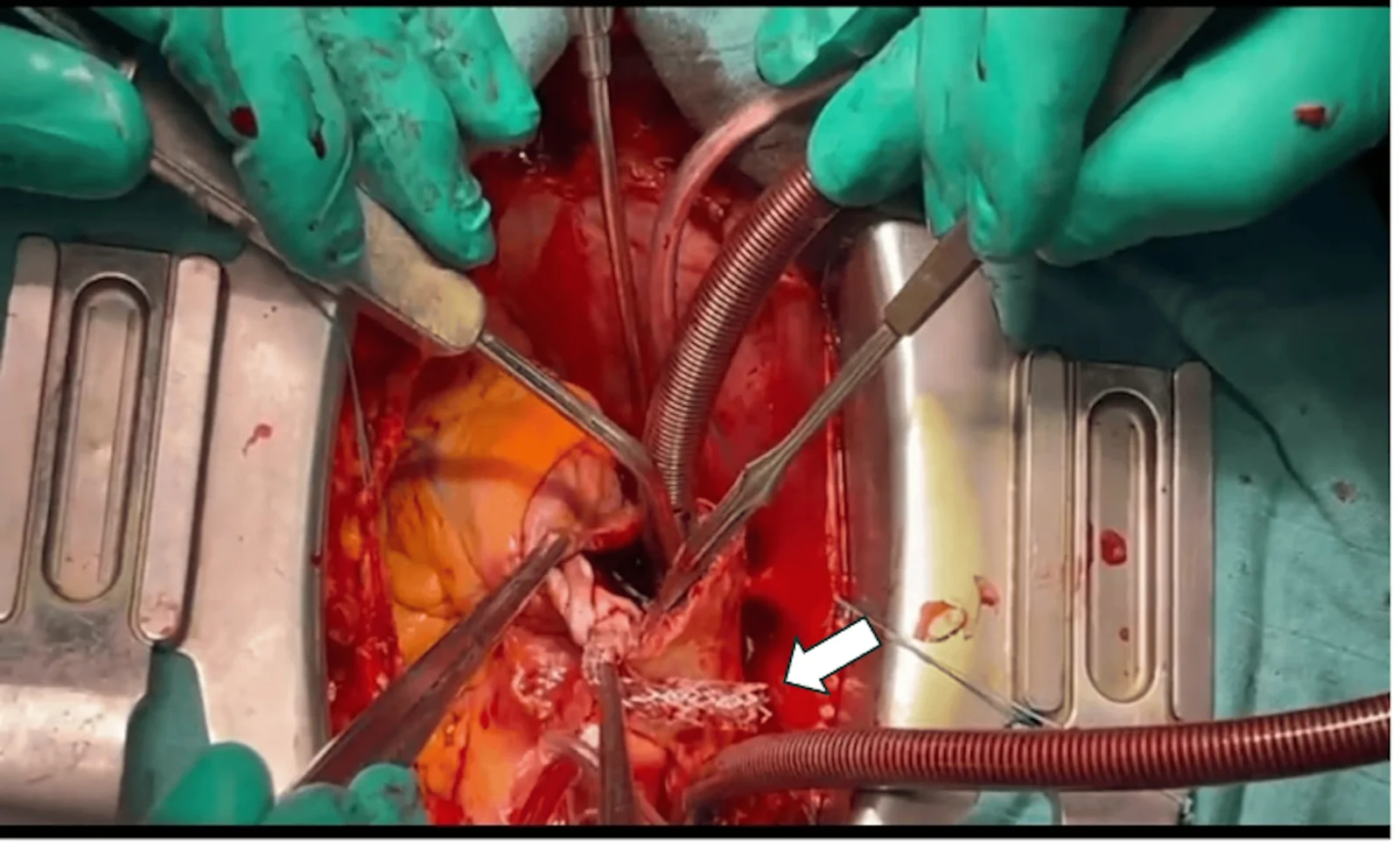
Surgical extraction of the migrated venous stent Intraoperative view during open-heart surgery under cardiopulmonary bypass showing extraction of the migrated venous stent from the right-sided cardiac chambers. The arrow indicates the stent during surgical removal.

Given the intracardiac position of the stent, its close relationship with the tricuspid valve apparatus, and the associated moderate tricuspid regurgitation, extraction was indicated. An endovascular retrieval strategy was considered unsuitable because the stent appeared to be embedded within the tricuspid valve apparatus, with a potential risk of worsening valvular injury or causing embolization. The patient therefore underwent surgical extraction through median sternotomy under cardiopulmonary bypass (Figure 4). A right atriotomy was performed, and the migrated stent was carefully disengaged from the tricuspid valve apparatus and completely removed. The cardiopulmonary bypass time was 70 minutes. The procedure was performed on the beating heart without aortic cross-clamping.

The postoperative course was uncomplicated, and the patient was discharged on postoperative day nine. He was clinically stable at discharge. At the three-week follow-up, the patient remained asymptomatic and in good general condition, with no clinical signs of recurrent right-sided heart failure.

## Discussion

Venous stenting is an established treatment option in selected patients with chronic venous obstruction. It is not routinely indicated after all cases of deep venous thrombosis but may be considered in patients with persistent disabling symptoms, severe post-thrombotic syndrome with residual venous obstruction, chronic iliac-caval thrombosis, or extrinsic iliac vein compression [1,2]. According to the 2020 appropriate use criteria, iliac vein or inferior vena cava stenting may be considered appropriate in selected symptomatic patients with advanced chronic venous disease, particularly those classified as CEAP C4-C6, in the absence of significant superficial venous reflux and when intravascular ultrasound demonstrates at least 50% iliac or caval stenosis or occlusion [3]. In addition, the 2014 Cardiovascular and Interventional Radiological Society of Europe (CIRSE) standards of practice guidelines suggest that patients with chronic venous outflow obstruction and CEAP clinical classes C3-C6 should be considered for interventional treatment [9]. Endovascular treatment with angioplasty and stent placement has become an established approach for restoring and maintaining venous outflow in patients with iliofemoral venous obstruction, with high technical success and acceptable complication rates across different causes of obstruction [10]. In the present case, the patient had undergone left iliac venous stenting approximately two decades earlier because of persistent symptoms following deep venous thrombosis.

Although venous stenting is considered safe in most cases, several complications have been described. These include thrombosis, restenosis related to intimal hyperplasia, infection, pseudoaneurysm formation, fracture, and migration [4,5]. Stent migration is rare but may have severe consequences when the device reaches the right heart or pulmonary circulation [5-8]. Several factors may contribute to migration, including inappropriate stent sizing, inadequate expansion, poor anchoring, unsuitable stent type, discrepancy between the stent and venous diameter, and changes in venous flow dynamics [4,5]. A stent diameter smaller than the target vessel may increase the risk of displacement. Therefore, careful preprocedural assessment, appropriate device selection, and accurate deployment are essential to reduce the risk of migration. Contemporary dedicated venous stents, including the Venovo, Abre, and Vici systems, are self-expanding nitinol devices specifically designed to accommodate the compliance and biomechanical forces of the iliofemoral venous system [11]. However, the exact manufacturer and model of the stent implanted in our patient approximately 20 years earlier could not be retrieved from the available medical records.

Intracardiac migration may be discovered incidentally during imaging performed for unrelated symptoms, as in our patient, or it may present with cardiac manifestations. The clinical presentation depends on the final position of the migrated stent and its relationship with intracardiac structures. Potential complications include tricuspid regurgitation, arrhythmias, right-sided heart failure, right ventricular perforation, endocarditis, and pulmonary embolization [6-8]. In our case, the stent was positioned across the right atrium and right ventricle, directly contacting the tricuspid valve apparatus and causing moderate tricuspid regurgitation with clinical signs of right-sided heart failure.

The management of intracardiac stent migration should be individualized. Conservative management may be considered in selected asymptomatic patients when the stent is stable and does not cause hemodynamic compromise or structural damage. However, extraction should be considered when the migrated device is associated with symptoms, valvular dysfunction, arrhythmia, perforation, infection, or risk of embolization [5,6]. Retrieval may be performed using either an endovascular or surgical approach. Endovascular retrieval can be attempted using snares, balloon catheters, or specialized extraction sheaths through femoral or jugular venous access, particularly when the stent is freely mobile and not embedded in cardiac structures [4,12]. In contrast, open surgical extraction is preferred when the stent is entangled in the tricuspid valve apparatus, embedded in the myocardium, associated with perforation, or considered unsuitable for safe percutaneous retrieval [6,7]. In cases of complex entanglement with the tricuspid valve apparatus, forceful or blind percutaneous traction may cause injury to the valve leaflets, subvalvular apparatus, or right ventricular wall, potentially resulting in irreversible tricuspid dysfunction. Surgical extraction under direct visualization and cardiopulmonary bypass may therefore provide a more controlled and safer approach in selected cases [13].

A systematic review by Sayed et al. highlighted that venous stent migration is an uncommon event that may nevertheless lead to serious clinical consequences and requires individualized management according to the location of the stent, associated symptoms, and feasibility of retrieval [5]. In our patient, surgical extraction was selected because the stent was closely related to the tricuspid valve apparatus and was responsible for clinically relevant tricuspid regurgitation. Attempted percutaneous retrieval could have worsened valvular injury or caused embolization. Open-heart extraction under cardiopulmonary bypass allowed safe removal of the foreign body and avoided further damage to the tricuspid valve.

This case emphasizes several important lessons. First, venous stenting should be reserved for appropriate indications and performed after careful clinical and anatomical assessment [1-3,9]. Second, correct stent sizing, appropriate device selection, and accurate deployment are essential to prevent migration [4,5,11]. Third, long-term surveillance may be important, as migration can be delayed and discovered years after the initial procedure [5]. Surveillance should be individualized according to the underlying venous disease, clinical symptoms, and imaging findings, with prolonged monitoring particularly in patients with post-thrombotic obstruction. Finally, once intracardiac migration is diagnosed, early multidisciplinary assessment involving cardiology, vascular surgery, interventional radiology, and cardiac surgery is necessary to determine the safest retrieval strategy [5].

## Conclusions

Intracardiac migration of an iliac venous stent is a rare but potentially serious complication of venous stenting. When the migrated stent reaches the right-sided cardiac chambers, it may interfere with the tricuspid valve and cause clinically significant tricuspid regurgitation or right-sided heart failure. Echocardiography and computed tomography are essential for diagnosis, anatomical assessment, and therapeutic planning. Management should be individualized according to the location of the stent, its mobility, its relationship with cardiac structures, and the patient’s clinical status. In cases of tricuspid valve entanglement, open-heart surgical extraction under cardiopulmonary bypass may be the safest and most effective option.
